# Validity and responsiveness of EQ-5D-5L and SF-6D in patients with health complaints attributed to their amalgam fillings: a prospective cohort study of patients undergoing amalgam removal

**DOI:** 10.1186/s12955-021-01762-4

**Published:** 2021-04-17

**Authors:** Admassu N. Lamu, Lars Björkman, Harald J. Hamre, Terje Alræk, Frauke Musial, Bjarne Robberstad

**Affiliations:** 1 Section for Ethics and Health Economics, Department of Global Public Health and Primary Care, Faculty of Health Sciences, University of Bergen, 5020 Bergen, Norway; 2 Dental Biomaterials Adverse Reaction Unit, NORCE Norwegian Research Centre AS, Bergen, Norway; 3 Department of Clinical Dentistry, University of Bergen, Bergen, Norway; 4 Institute for Applied Epistemology and Medical Methodology, University of Witten/Herdecke, Freiburg, Germany; 5 National Research Center in Complementary and Alternative Medicine, NAFKAM, Department of Community Medicine, UiT The Arctic University of Norway, Tromsø, Norway

**Keywords:** EQ-5D-5L, SF-6D, Utility, Responsiveness, Validity, Minimally important change

## Abstract

**Background:**

Evidence of health utility changes in patients who suffer from longstanding health complaints attributed to dental amalgam fillings are limited. The change in health utility outcomes enables calculating quality-adjusted life-year (QALY) and facilitates the comparison with other health conditions. The purpose of this study was to estimate the validity and responsiveness of the EQ-5D-5L and SF-6D utilities following removal of dental amalgam fillings in patients with health complaints attributed to their amalgam fillings, and examine the ability of these instruments to detect minimally important changes over time.

**Methods:**

Patients with medically unexplained physical symptoms, which they attributed to dental amalgam restorations, were recruited to a prospective cohort study in Norway. Two health state utility instruments, EQ-5D-5L and SF-6D, as well as self-reported general health complaints (GHC-index) and visual analogue scale (EQ-VAS) were administered to all patients (n = 32) at baseline and at follow-up. The last two were used as criteria measures. Concurrent and predictive validities were examined using correlation coefficients. Responsiveness was assessed by the effect size (ES), standardized response mean (SRM), and relative efficiency. Minimally important change (MIC) was examined by distribution and anchor-based approaches.

**Results:**

Concurrent validity of the EQ-5D-5L was similar to that of SF-6D utility. EQ-5D-5L was more responsive than SF-6D: the ES were 0.73 and 0.58 for EQ-5D-5L and SF-6D, respectively; SRM were 0.76 and 0.67, respectively. EQ-5D-5L was more efficient than SF-6D in detecting changes, but both were less efficient compared to criteria-based measures. The estimated MIC of EQ-5D-5L value set was 0.108 and 0.118 based on distribution and anchor-based approaches, respectively. The corresponding values for SF-6D were 0.048 and 0.064, respectively.

**Conclusions:**

In patients with health complaints attributed to dental amalgam undergoing amalgam removal, both EQ-5D-5L and SF-6D showed reasonable concurrent and predictive validity and acceptable responsiveness. The EQ-5D-5L utility appears to be more responsive compared to SF-6D.

*Trial registration* The research was registered at ClinicalTrials.gov., NCT01682278. Registered 10 September 2012, https://clinicaltrials.gov/ct2/show/NCT01682278.

## Background

Dental amalgam is one of the most widely used dental restorative materials, but safety concerns relating to its mercury content have been a topic of discussion for many years [1]. This has been a particularly contentious issue in Norway, where amalgam use was banned in 2008, due to environmental considerations. Some people with amalgam fillings report health complaints with a pattern similar to patients with medically unexplained physical symptoms (MUPS), where the symptoms are non-specific and a known pathophysiology is missing [2, 3]. Furthermore, they attribute their health complaints to mercury released from their amalgam fillings [3]. Hereafter we refer to these patients as *amalgam patients*. Thus, assessing health outcomes in amalgam patients and their health-related quality of life is essential for clinical practice and future research strategies. Evidence of health utility changes and their validity and responsiveness in patients who had longstanding health complaints attributed to dental amalgam fillings is sparse. Thus, additional studies of the change in health utility outcomes that enable the calculation of quality-adjusted life year (QALY) and facilitate the comparison with other health conditions are warranted.

Health state utility (HSU) instruments consist of a descriptive system and a predetermined utility weight. Utilities are cardinal values that reflect an individual’s preferences assigned to each possible combination of health states in the descriptive system [4, 5]. They are measured on an interval scale with zero reflecting states of health equivalent to death and one reflecting full health. The EuroQol Five-Dimensional Questionnaire (EQ-5D) [6] and the Medical Outcomes Short-Form Six-Dimension (SF-6D) [7] are the most widely used HSU instruments. Such utilities are typically combined with survival estimates and aggregated across individuals to generate QALYs. QALYs are widely used as a measure of health outcomes in economic evaluations of health interventions. Economic evaluations play an increasing role in resource allocation decisions in healthcare, and it is important to critically assess the utility weights that form the basis for estimating QALY [8].

The EQ-5D has five dimensions, each with three severity levels in the original version (EQ-5D-3L) and five severity levels in the revised version (EQ-5D-5L). The revised version was designed to minimize the ceiling effect and improve the sensitivity of the 3L version [9]. There are several country-specific EQ-5D-5L valuation studies that are currently ongoing (including the elicitation of Norwegian values) or already published [10]. The EQ-5D instrument is commonly recommended for economic evaluation by reimbursement agencies, such as the National Institute for Care and Excellence (NICE) in the United Kingdom (UK) [11] and the Norwegian Medicines Agency in Norway [12]. Despite the publication of the new English EQ-5D-5L value set, NICE’s interim-position at the time of the analysis (December 2020) is that the validated cross-walk or mapping function by van Hout et al. [13] to derive value sets for the EQ-5D-5L from the existing 3L version should be used for economic evaluation. Based on NICE’s recommendation, the Norwegian Medicines Agency also use the cross-walk value sets by van Hout and colleagues for single technology assessments [12]. In general, EQ-5D is the most widely used HSU instrument in economic evaluation, followed by SF-6D [14, 15], and hence the focus of the present study.

The psychometric properties of EQ-5D and SF-6D have been widely investigated in different patient groups as well as in the general population. A recent systematic review of the literature demonstrated excellent psychometric properties of the EQ-5D across a broad range of populations and conditions [16]. The EQ-5D appears to be reliable and valid in the general population [17–22], and so does the SF-6D [23–25]. Thus, the reliability and validity of such instruments in the general population are useful for future population health studies. In addition to evidence of validity, both EQ-5D and SF-6D have been shown to be responsive in the general population, but the mean quality of life measured by EQ-5D is usually higher than that of SF-6D [26–28].

Furthermore, both EQ-5D and SF-6D have shown evidence of validity, and responsiveness for a number of diseases [29–36]. One important test of validity is the ability of a health outcome measure to reflect relevant changes in the health of patients over time, which, specifically, is referred to as responsiveness of a measure [37]. Although these instruments are becoming more common in clinical practice, the meaning of a change in score on such HSU instrument is not straightforward, mainly because of the unfamiliar units in the scale of these instruments [38]. These health outcome measures can be completed at baseline and at follow-up, and generate score changes, which allows us to easily calculate the statistical significance of the score changes. However, establishing the magnitude of the change score in a way easily understandable for health professionals, patients and policy-makers has been difficult, though not impossible [38, 39]. Quantifying the magnitude of change that corresponds to a minimally important difference would help to address this problem [40]. From the patient perspective, Jaeschke et al. [41] defined the minimally important difference as “the smallest difference in score in the domain of interest which patients perceive as beneficial”. Although we have some knowledge about validity and responsiveness of EQ-5D and SF-6D in some diseases [31, 35, 38, 42–44], we know very little about their responsiveness and validity in patients with MUPS who attribute their health problems to dental amalgam. To our knowledge, the minimally important difference of EQ-5D and SF-6D for *amalgam patients* has not been reported so far.

Therefore, we aimed to examine the health utility changes associated with dental amalgam fillings removal in a group of *amalgam patients* in terms of the two most widely used HSU instruments; the *EQ-5D-5L* and *SF-6D*. More specifically, this analysis had two objectives: (i) examine the concurrent and predictive validity as well as responsiveness to change of the EQ-5D-5L and SF-6D utilities in a prospective cohort study; and (ii) assess the ability of the EQ-5D-5L and SF-6D instruments to detect minimally important changes over time.

## Methods

### Data and study design

The analyses are based on data from a prospective cohort in Norway [45]. The study comprised three groups recruited separately: (i) patients with MUPS, which they attributed to dental amalgam restorations who wished to have their amalgam fillings removed (Amalgam cohort); (ii) patients with MUPS recruited from general practice without symptom attribution to amalgam fillings (MUPS cohort); and (iii) participants who identified themselves as healthy (Healthy cohort).

This analysis is restricted to the Amalgam cohort, which was the main target group. The presence of at least one amalgam fillings is the primary criteria for inclusion in amalgam cohort. Other inclusion criteria were unspecific health complaints attributed to dental amalgam restorations at least for three months, wish to have all amalgam fillings removed, ability to comply with the protocol, age between 20 and 70 years, permanent residence in Norway. Detailed eligibility criteria and recruitment procedures are reported elsewhere [45]. In general, a total of 49 participants were initially assessed for inclusion in the Amalgam cohort. Of these, 12 subjects did not fulfill the eligibility criteria and 5 did not complete the amalgam removal, and were excluded from the analysis. A total of 32 participants were available for the follow-up analysis.

### Variables

At baseline, the socio-economic variables age, gender, marital status, education, household income and employment status were documented. Patients also completed the EQ-5D-5L and the short-form 36 questionnaires (SF-36) at baseline and at follow-up (12 months after amalgam removal). Based on SF-36 questionnaire, we calculated the SF-6D utility [7]. Patients also rated their overall health on the visual analogue scale (EQ-VAS) and documented general symptoms in a health complaints index (GHC-index) questionnaire. The latter two (GHC and EQ-VAS) were used as the criteria variable against which the utility instruments were compared.

#### Europol Five-Dimensional Questionnaire (EQ-5D-5L)

At the time of this analysis, the psychometric property of the EQ-5D-5L was not yet studied in Norwegian population. However, a study of the Norwegian population norms for EQ-5D-3L [46] demonstrated that it can be used as reference data to compare patients with specific conditions and to assess the burden of the condition in question. The EQ-5D-5L^1^ describes health along five dimensions: mobility, self-care, usual activities, pain/discomfort and anxiety/depression. Each dimension is assessed by a single question on a five-point ordinal scale (no problems, slight problems, moderate problems, severe problems, extreme problems/unable to). Thus, the EQ-5D-5L defines 5^5^ = 3125 possible *health states*. These health states can be converted to a single EQ-5D-5L summary index by applying scores from a standard set of values (utility weights) derived from general population samples [47]. In the absence of Norwegian utility weights, we used utility weights that were derived from members of the English general public using composite time-trade-off [48]. We also tested the consistency of our results by applying the United Kingdom (UK) utility weights calculated using cross-walk algorithm (mapping the EQ-5D-5L descriptive system data onto the 3L value set) from van Hout et al. [13]. The UK cross-walk value set is denoted as EQ-5D-CW to distinguish it from the directly elicited English value set, defined as EQ-5D-5L. The minimum value for the worst health state (“the pits”) was − 0.285 for the English EQ-5D-5L value set and -0.594 for the UK EQ-5D-CW.

The full health state, which is the absence of any problem on each of the 5-dimensions of the EQ-5D-5L (11111), gives a utility of 1. In contrast, the worst health state—which corresponds to level-5 on each dimension (55555); i.e., unable to walk about, unable to wash or dress myself, unable to do my usual activities, have extreme pain/discomfort, and extremely anxious/depressed—produces a negative utility. The exact value of health state utilities vary depending on which country’s value set (utility weight) has been applied [8]. In general, negative utilities imply that patients would prefer to die than live with such poor health states. Thus, utility is the preference for a health state (rated in the presence of choice) relative to full health (scored 1) and death (scored 0), and values below zero representing health states worse than being dead.

#### The Short-Form Six-Dimension (SF-6D)

The SF-6D^2^ is derived from 11 items of the SF-36 or SF-12 health survey, and has six-dimensions: physical functioning, role functioning, social functioning, bodily pain, mental health, and vitality [7, 49]. Each dimension has four to six severity levels, defining 18 000 unique health states. Studies for the population norms of the SF-36 in the Norwegian general population supported the validity of the instrument [50, 51]. Since there is no Norwegian-specific SF-6D value sets, the utility weights for SF-6D health states are based on members of the UK general population, and were elicited using standard gamble [7]. The maximum SF-6D utility is 1, the minimum score for a living person (the worst state) is 0.296, and the state being *dead* is scored as zero.

#### EuroQol visual analogue scale (EQ-VAS)

For the EQ-VAS, which is part of the EQ-5D-5L questionnaire, patients are asked to indicate their overall health on a vertical visual analogue scale, ranging from 0 (*worst imaginable health*) to 100 (*best imaginable health*). EQ-VAS can be used to measure a multitude of subjective conditions and would seem particularly appropriate for conditions related to MUPS.

#### General health complaints (GHC-index)

The GHC-index is the sum score of 12 items, each scored by use of numeric rating scales ranging from 0 to 10 [52]. The items are: Musculoskeletal complaints, gastrointestinal complaints, cardiovascular complaints, skin problems, complaints related to eyes/sight, complaints related to ears/hearing/nose/throat, tiredness, dizziness, headaches, memory problems, difficulty concentrating, and anxiety/depression. We reversed the score values (GHCr) to obtain a scale in which higher scores represent better health (i.e., less health complaints) and positive change scores represented improvement. The GHC-index is not utility-weighted, and the index value is the crude sum of scores over the 12 dimensions with a maximum value of 120.

### Statistical analyses

#### Concurrent and predictive validity

Concurrent validity of each of the two HSU instruments was tested by computing Spearman’s rank correlation coefficients (*rho, ρ*) between the utility instruments and each of the criterion measures at the baseline. A non-parametric Spearman’s rank correlation coefficient was chosen based on the measures’ score distributions. Because health is not a static variable, we repeated the concurrent validity analyses in the follow-up period.

Predictive validity refers to the association between one variable and an outcome assessed at a later time [53, 54]. In this study, predictive validity was calculated as the correlation (*ρ*) between the HSU instruments at baseline and the criterion measures at follow-up. The strength of the relationship was considered low/weak (*ρ* < 0.25), fair (*ρ* = 0.25–0.50), good (*ρ* = 0.50–0.75), and excellent (*ρ* > 0.75) [16].

#### Responsiveness

Responsiveness is a measure of the sensitivity of an instrument to change in health status over time. First, changes in all health outcome measures at the baseline and follow-up were compared using paired *t*-tests. Second, responsiveness was assessed using effect size (ES), standardized response mean (SRM) and relative efficiency (RE). ES was defined as the mean observed change from baseline to follow-up divided by the standard deviation of the baseline score [55]. SRM was calculated in the same way as the ES, but using the standard deviation of the pre-post differences as denominator. ES and SRM were classified as large (≥ 0.8), moderate (0.5–0.79) or small (< 0.5) [56, 57]. Both ES and SRM are standardized measures of change over time in health, independent of sample size [58]. RE was calculated by taking a ratio of *F*-statistics (or squared *t*-statistics), where the criterion measure served as the reference. An RE value less than 1 implies that the standard criterion measure is more responsive than the utility instruments, and the converse is true for an RE value of greater than 1. A coefficient of 1 indicates similarity in the efficiency of the two measures.

#### Minimally important change

Minimally important change (MIC) is defined as the smallest change in score which is perceived as important by patients or clinicians [59]. In this study, MIC was assessed using both distribution and anchor-based approaches. Distribution-based methods mainly measure minimally detectable change (the smallest change that can be detected by the instrument beyond measurement error) [60]. Nonetheless, the term MIC is used for both approaches in this paper. For distribution-based calculations, MIC was defined as half baseline standard deviation (0.5*SD) for the effectiveness of the intervention [61]. For the anchor-based method, MIC is usually estimated by comparing change scores with an external anchor. One of the commonly used anchors for establishing MIC is global ratings of change. For the present analysis, we used patients’ self-reported global ratings of results of amalgam fillings removal as external anchor. Participants were asked “How do you rate the results of the amalgam fillings removal?” The pre-defined responses were:

1. Fully recovered.
2. Much better.
3. Somewhat better.
4. No change.
5. Worse.

The relationship between the global ratings of change question and changes in EQ-5D-5L and SF-6D utilities was assessed by calculating the change in EQ-5D-5L and SF-6D utilities from baseline to follow-up for each patient, and likewise for the GHCr-index and EQ-VAS. In accordance with previous studies, we considered patients whose global ratings of change score was 3 or 5 as having experienced some change equivalent to the MIC [38, 62]. For participants who reported a worsening of health (rating scale of 5), the sign of the change in each health outcome measure was reversed. The MIC was then taken as the mean changes in scores of the patients who scored 3 or 5.

Finally, we applied predictive modelling, which is a newly proposed anchor-based method for MIC [63]. Here, Item-2 of the SF-36 was used as the anchor, which is described as: “Compared to one year ago, how would you rate your health in general now?”.

1. Much better now than 1 year ago.
2. Somewhat better now than 1 year ago.
3. About the same.
4. Somewhat worse now than 1 year ago.
5. Much worse now than 1 year ago.

This score was transformed into a change score by taking the difference between baseline and follow-up to produce an anchor. Then, we used *exact* logistic regression to predict whether a patient belongs to the improved (≥ 1) or not improved group (≤ 0) on this anchor using the change in each of the health outcome measure as the predictor. When sample sizes are small or the data are skewed, *exact* conditional inference is often more appropriate compared to the conventional method of logistic regression [64]. The MIC was estimated from the equation: $MIC=\left[\mathrm{ln}\left({Odds}_{Pre}\right)-C\right]/\beta$, where C is the intercept, β is the regression coefficient, and Odds_Pre_ is pre-odds of being improved and given by P/(1 − P), with P the proportion improved based on the anchor [63].

To confirm the usefulness of the anchors, Spearman’s rank correlations were computed between the health outcome score changes and the two anchors. A correlation coefficient of 0.30 or more is considered sufficiently strong to allow for computation of MIC [65].

## Results

### Baseline characteristics

**Fig. 1:**
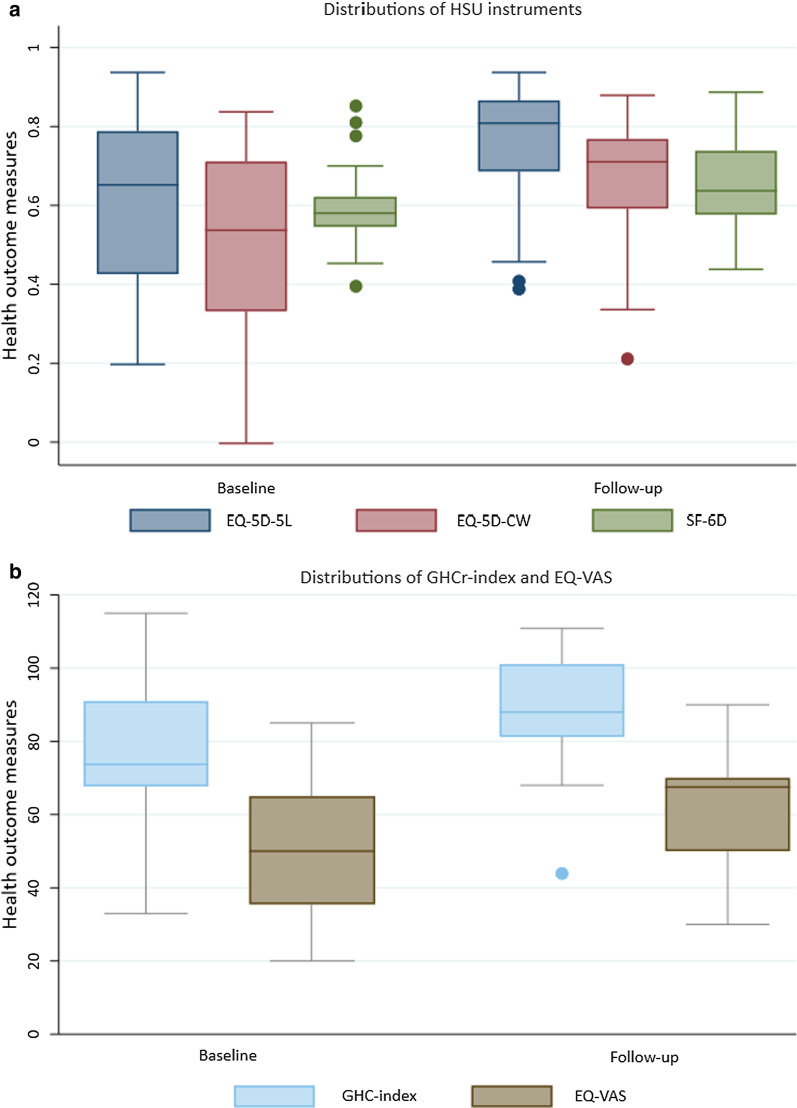
Box plots showing distributions of health outcomes at the baseline and follow-up for amalgam patients. A box indicates the positions of the upper and lower quartiles; the interior of the box indicates the interquartile range; the crossbar (middle line) that intersects the box shows the median of the dataset; a whisker (line) that extends to the extreme of the distribution from lower hinge and upper hinge indicates the minimum and maximum values, respectively. *EQ-5D-5L* EuroQol 5-dimensional 5-level questionnaire; *EQ-5D-CW* EQ-5D cross-walk value set; *SF-6D* Short-form 6-dimension; *GHCr* (reversed) general health complaints; *EQ-VAS* (EuroQol) visual analogue scale; *HSU* Health state utility

Demographic and socio-economic characteristics are presented in Table 1. Most patients were female (59.4%), with the majority living with a partner or spouse (81.3%). Mean age of patients at baseline was 52 (SD = 7.5) years. Boxplots illustrate that the distributions were wide for all five health outcomes measures used, both at baseline and at follow-up (Fig. 1). No patient reported either the worst or best possible health on any of the health outcome measures**.**

**Table 1:** Baseline sample characteristics for patients with MUPS undergoing amalgam removal

Characteristics	N (%)
Female gender	19 (59.4)
Education	
Lower & upper secondary	14 (43.8)
College, < 4 years	11 (34.4)
College, 4 + years	7 (21.9)
Living with partner	
No	6 (18.7)
Yes	26 (81.3)
Income	
Low	9 (25.8)
Middle income	13 (41.9)
High income	10 (32.3)

*MUPS* medically unexplained physical symptoms

### Concurrent and predictive validity

Spearman’s correlation coefficients for the HSU instruments and the criteria measures (GHCr-index and EQ-VAS) at baseline and follow-up periods are presented in Table 2. For the EQ-5D-5L, the correlations with GHCr-index were relatively lower at baseline (ρ = 0.48) than at follow-up (ρ = 0.52), with a reverse pattern for EQ-VAS (higher at baseline). The results were quite similar for the EQ-5D-CW and the SF-6D. These correlations were generally high, indicating good concurrent validities of the HSU instruments. With regard to predictive validity, the EQ-5D-5L and SF-6D at the baseline period predicted both the GHCr-index and EQ-VAS at follow-up period *fairly* well, with SF-6D performing slightly better. The EQ-5D-CW fairly predict EQ-VAS, but slightly week in predicting GHCr-index.

**Table 2:** Correlations as measures of concurrent and predictive validities for EQ-5D-5L and SF-6D utilities

	EQ-5D-5L	EQ-5D-CW	SF-6D
*Concurrent validity*			
Baseline			
GHCr-index	0.48	0.46	0.42
EQ-VAS	0.74	0.68	0.70
Follow-up			
GHCr-index	0.52	0.56	0.50
EQ-VAS	0.55	0.58	0.64
*Predictive validity*^*a*^			
GHCr-index	0.25	0.22	0.31
EQ-VAS	0.31	0.28	0.46

^a^Correlations between utility instruments at baseline and criteria variables at follow-up, which are classified as weak (*ρ* < 0.25), fair (0.25 to 0.50), good (0.50 to 0.75), and excellent (> 0.75). *EQ-5D-5L* EuroQol 5-dimensional 5-level questionnaire; *EQ-5D-CW* EQ-5D cross-walk value set; *SF-6D* Short-form 6-dimension; *GHCr* (reversed) general health complaints; EQ-VAS, (EuroQol) visual analogue scale

### Responsiveness and minimally detectable change

Responsiveness results are presented in Table 3. The baseline means (SD) EQ-5D-5L and SF-6D utilities were 0.61 (0.22) and 0.60 (0.10), respectively. Mean EQ-5D-CW was 0.10 lower than mean EQ-5D-5L both in the baseline and follow-up. All health outcome measures showed significant improvement at follow-up (*p* < 0.01, paired *t*-tests). The mean change (SD) for EQ-5D-5L was 0.158 (0.207) and that of SF-6D was 0.056 (0.084). The mean change for the EQ-5D-CW is quite like the EQ-5D-5L. The corresponding values for GHCr-index and EQ-VAS were 12.78 (15.87) and 12.53 (15.23), respectively.

**Table 3:** Measures of responsiveness for health outcome measures

	Mean (SD)		Paired t-test		Responsiveness		
	Baseline	Follow-up	∆Mean (SD)	*p* value	ES (SE)	SRM (SE)	RE (SE)
EQ-5D-5L	0.61(0.22)	0.77 (0.16)	0.158 (0.207)	< 0.001	0.73 (0.17)	0.76 (0.18)	0.90 (0.58)
EQ-5D-CW	0.51(0.23)	0.67 (0.17)	0.162 (0.220)	0.002	0.70 (0.16)	0.75 (0.19)	0.84 (0.57)
SF-6D	0.60 (0.10)	0.65 (0.12)	0.056 (0.084)	0.001	0.58 (0.19)	0.67 (0.23)	0.68 (0.34)
GHCr-index^a^	76.72 (17.75)	89.49 (14.36)	12.78 (15.87)	< 0.001	0.72 (0.19)	0.81 (0.18)	1 (Ref.)
EQ-VAS	49.90 (18.21)	62.44 (16.72)	12.53 (15.23)	< 0.001	0.69 (0.17)	0.82 (0.19)	1.05 (0.48)

^a^ GHCr-index has been used as a reference in the calculation of RE. *ΔMean* Mean change between baseline and follow-up; *SD* standard deviation; *SE* bootstrapped standard error (with 1000 iterations); *ES* effect size; *SRM* standardized response mean; *RE* relative efficiency; *EQ-5D-5L* EuroQol 5-dimensional 5-level questionnaire; *EQ-5D-CW* EQ-5D cross-walk; *SF-6D* Short-form 6-dimension; *GHCr* (reversed) general health complaints; *EQ-VAS* (EuroQol) visual analogue scale

Generally, all health outcome measures exhibited *moderate* responsiveness as measured by ES and SRM. Except the SF-6D, all health outcome measures revealed similar ES (≈ 0.70). The SF-6D utility instrument had relatively lower responsiveness (ES = 0.58) (Table 3). In terms of SRM, both criteria variables revealed *large* responsiveness (SRM > 0.80), while the two utility instruments indicated *moderate* responsiveness, as expected. All HSU instruments had RE below 1, indicating less efficiency in detecting changes in health over time as compared to GHCr-index (though statistically insignificant, except SF-6D). Both EQ-5D-5L and EQ-5D-CW were more efficient when compared with SF-6D.

MIC estimations derived from the anchor- and distribution-based methods, as well as the correlations between health outcome score changes and the two anchors are presented in Table 4. All health outcome score changes were significantly correlated with responses to both anchors; accordingly, the two global ratings of change were considered appropriate anchors for measuring all score changes.

**Table 4:** Minimally important changes for those who reported some change

	Correlations (ρ)^a^		0.5 SD	Anchor-based method		Predictive modelling	
	GRC1	GRC2	MIC	MIC	95% CI	MIC	95% CI
EQ-5D-5L	0.53	0.49	0.108	0.118	0.038–0.198	0.103	0.062–0.237
EQ-5D-CW	0.52	0.45	0.115	0.124	0.036–0.213	0.101	0.018–0.325
SF-6D	0.45	0.51	0.048	0.064	0.029–0.099	0.056	0.020–0.143
GHCr-index	0.36	0.31	8.876	9.158	1.798–16.517	8.786	3.096–17.804
EQ-VAS	0.45	0.36	9.106	8.789	2.580–14.999	9.076	3.525–18.624

^a^ Correlations of GRC variables with score changes of utility and criteria variables (a value of ρ 0.30 and above is sufficiently large to use the anchors for the calculation of MIC). *GRC* Global rating of change (1 = evaluation of amalgam removal results, & 2 = item-2 of the SF-36); *CI* (bootstrapped) confidence interval (with 1000 iterations); *SD* (baseline) standard deviation; *MIC* minimally important change; *EQ-5D-5L* EuroQol 5-dimensional 5-level questionnaire; *SF-6D* Short-form 6-dimension; *GHCr* (reversed) general health complaints; *EQ-VAS* (EuroQol) visual analogue scale

For the anchor-based method, the MIC estimation was 0.118 for the EQ-5D-5L, 0.124 for EQ-5D-CW and 0.064 for SF-6D. The corresponding MIC was 9.158 for the GHCr-index and 8.789 for the EQ-VAS score. The distribution-based estimation (i.e., half SD of baseline) gave 0.108 for the EQ-5D-5L, 0.115 for EQ-5D-CW and 0.048 for the SF-6D. Both the distribution- and anchor-based estimations produced fairly similar results. The predictive modelling approach also produced similar findings, confirming the consistency of our results.

## Discussion

In this analysis of patients with health complaints attributed to their amalgam fillings, validity and responsiveness to change from baseline to follow-up (12 months after removal of amalgam fillings) was assessed. This study is the first to assess the validity and responsiveness of two commonly used utility instruments—the EQ-5D-5L and SF-6D—in patients with MUPS who attribute their health problems to amalgam fillings. Our results have shown key differences in the ability of the EQ-5D-5L and SF-6D to measure longitudinal changes. Although both EQ-5D-5L and SF-6D demonstrated significant change in health over time, the EQ-5D-5L was more responsive to change than the SF-6D. This finding is in line with previous studies of the two utilities in other health conditions [31, 38, 66].

The correlation between the utility and criterion measures showed *fair* concurrent validity at both baseline and follow-up. The high correlations between criteria measures and the two HSU instruments generally demonstrated acceptable concurrent validity of EQ-5D-5L and SF-6D. At the end of the follow-up period, correlations were increased between the SF-6D and criterion measures unlike EQ-5D-5L. The main explanation could be that SF-6D captures most of the description of the more specific and comprehensive scale of GHCr-index and EQ-VAS compared to the EQ-5D-5L. For instance, both SF-6D and GHCr-index focus on physical functioning and energy, which the EQ-5D-5L lacks. This implies that the intervention was followed by reduction of intensity of the health complaints, increasing its consistency with the more specific and comprehensive scales of criteria measures [31].

Further, our findings showed that EQ-5D-5L and SF-6D had sufficient predictive validity—the utility instruments at the baseline would *fairly* predict the GHCr-index at follow-up, indicating that they accounted for a significant amount of variance in predicting amalgam treatment outcomes at follow-up. The predictive power may depend on the severity of the condition, which could not be investigated due to small sample size. However, regression to the mean could partly be considered as a possible cause of an observed change, because regression to the mean in repeated data is a ubiquitous phenomenon [67, 68].

With regard to responsiveness, our results generally showed *moderate* to *large* responsiveness for all the health outcome measures. The EQ-5D-5L and SF-6D were moderately responsive to changes based on both the ES and SRM statistics, whereas the EQ-VAS and GHCr-index were highly responsive to changes on the SRM and relative efficiency statistics. Other studies in different diseases found similar results [31, 66]. The high responsiveness and greater efficiency would suggest that GHCr-index and EQ-VAS are suitable criteria measures for amalgam patients. However, a firm conclusion that the GHCr-index and EQ-VAS are more responsive than the HSU instruments as a measure of health in amalgam patients would be premature, because the findings are based on an analysis with a small sample size.

To our knowledge, empirical work has not been performed to assess the responsiveness and MIC of the EQ-5D-5L and the SF-6D in patients with MUPS attributed to amalgam fillings. Both the anchor-based and distribution-based approaches have shown similar MIC values. While the MIC values calculated by the distribution- and anchor-based methods were fairly similar for the EQ-VAS and the GHCr-index, a substantial difference was observed between the EQ-5D-5L and SF-6D. The MIC difference between the two utility instruments was mainly attributable to their scale difference. For instance, the effective scale length for the English EQ-5D-5L is 1.285 (i.e., from -0.285 to 1), whereas the effective scale length for the SF-6D is 0.704 (i.e., 0.296 to 1). The scale adjusted anchor-based MIC for EQ-5D-5L was 0.092 (= 0.118/1.285), which is equivalent to that of SF-6D (0.091 = 0.064/0.704). Similar results were observed for the distribution-based MIC. Thus, the scale difference mainly accounts for the difference in their MIC values. Our finding is consistent with previous studies for EQ-5D-3L and SF-6D [38]. This implies that it is not only the description of health but also the range of the instrument scale that is crucial in the assessment of MIC for the HSU instruments. While EQ-5D-5L applied time-trade-off for elicitation of utility weights, SF-6D used standard gamble. Thus, their difference arises primarily because of scale effect brought up by the methodological approach used to construct preference weights [69].

The EQ-5D-5L and SF-6D are designed to be utilized to calculate QALYs (measured in units of time) for the estimation of cost-effectiveness. Therefore, the validity of the instruments will translate into the validity for the cost-effectiveness estimates. Usually, the EQ-5D-5L tends to produce larger change scores than the SF-6D and hence produces more favorable cost-effectiveness ratios than the SF-6D, especially when baseline health was strongly compromised as the case in the present study [70]. Similarly, other studies found higher QALY gains using the EQ-5D-3L than the SF-6D [33, 36, 71, 72].

Both HSU instruments had good concurrent validity and fair predictive validity in patients with amalgam removal. While EQ-5D consistently showed better responsiveness, SF-6D had slightly better predictive power. The results also depend on the choice of the criteria variables and the disease conditions in question. Being more responsive, the EQ-5D could be more appropriate for measuring the burden of health conditions or for generating QALYs that can be used in economic evaluation studies than the SF-6D in line with previous study [73]. Nevertheless, both HSU instruments are valid economic evaluation instruments but not interchangeable and hence, the choice of HSU instrument for measuring utility can lead to different results in the context of cost per QALY estimates. This suggests that researchers and policy makers using cost-effectiveness analysis must consider several sources of evidence to select an instrument for measuring utility. Since there is no gold standard, decision makers need to consider an instrument that enable them to make consistent decisions across a broad range of populations and conditions. Further research is required, particularly across the full severity range of the utility scale, to identify the practical performance of utility instruments and their implications for cost-per-QALY estimates and health care decision making.

The main strength of this study is that it applied several techniques to validate instruments to elicit health outcome measures, including ES, SRM and relative efficiency as well as mean change scores to measure the responsiveness, which enables us to confirm the consistency of our results. Further, we applied distribution-based and multiple anchor-based assessment of MIC for the EQ-5D-5L and SF-6D that substantiate the stability of our findings. Although there is no single gold standard external criterion, anchor-based techniques rely most commonly on the use of a subjective global assessment of change [74], which have the advantage of linking the change in a given score to the patient’s perspective [58].

This study has also a number of limitations. Because global assessment is based on the recall ability of patients about their earlier health status, the use of the retrospective global assessment as an external criterion of score changes can be problematic. That is, there could be a potential for response shift and recall bias due to the prolonged time between the baseline and follow-up periods [38]. Thus, the MIC may change over time and recall bias and response shift may pose a problem, which needs to be investigated further. Furthermore, only a few patients reported deterioration and, hence, we did not analyse MIC for the clinically deteriorated patients separately. The small sample size also precluded the use of subgroup analyses. Thus, although this study has used several methods to quantify MIC, it is important to further test and validate estimates using other methods and larger sample sizes.

## Conclusions

In conclusion, the concurrent and predictive validity from all health outcomes were acceptable. The discrepancy in responsiveness of EQ-5D-5L and SF-6D in detecting change was mainly attributed to their scale differences. Thus, both EQ-5D-5L and the SF-6D can be used in clinical trials including this group of patients where a known effective intervention is to be applied. The MIC estimate for the EQ-5D-5L and SF-6D will be useful in interpreting EQ-5D-5L and SF-6D utilities, both in individuals and in groups of patients participating in trials as well as in the planning of new trials. The differences in the magnitude of the absolute change scores have important implications for cost-effectiveness analyses. Economic evaluation studies should be based on health utilities elicited with instruments that have validated measurement properties for the intended population.

## Data Availability

The datasets generated and analysed during the current study are not publicly available due to privacy concern as relatively few patients participated in the study with implications for potential identification through personal characteristics.

## Abbreviations

EQ-5D (5L/3L/CW): EuroQol 5-Dimensional (5-Level/3-level/Crosswalk)
EQ-VAS: EuroQol Visual Analogue Scale
ES: Effect Size
GHCr: General Health Compliant (reversed)
GRC: Global rating of change
HSU: Health State Utility
MIC: Minimally Important Change
MUPS: Medically Unexplained Physical Symptoms
NICE: National Institute for Care and Excellence
QALY: Quality-Adjusted Life Year
RE: Relative Efficiency
ρ: Spearman’s rank correlation coefficient (rho)
SD: Standard Deviation
SE: Standard Error
SF-6D: Short Form 6-Dimension
SF-36: Short Form 36 (questionnaire)
SRM: Standardized Response Mean
UK: United Kingdom

## Footnotes

1 EQ-5D-5L was available after registration at EuroQol Group http://www.euroqol.org.

2 SF-6D was available after registering at University of Sheffield https://licensing.sheffield.ac.uk/product/SF-6D.
